# Down-Klinefelter Syndrome (48,XXY,+21) in a Saudi Neonate: A Case Report and Literature Review

**DOI:** 10.7759/cureus.24561

**Published:** 2022-04-28

**Authors:** Jubara Alallah, Sohaib Habhab, Farzeen Mohtisham, Aiman Shawli, Mustafa Daghistani

**Affiliations:** 1 Pediatrics, Neonatology, King Saud Bin Abdulaziz University for Health Sciences College of Medicine, Jeddah, SAU; 2 Pediatrics, Neonatology, King Saud Bin Abdulaziz University for Health Sciences, Jeddah, SAU; 3 Pediatrics, Neonatology, King Khalid Medical City, National Guard Hospital, Jeddah, SAU; 4 Pediatrics, King Saud Bin Abdulaziz University for Health Sciences College of Medicine, Jeddah, SAU; 5 Pediatrics, King Abdulaziz Medical City Riyadh, Jeddah, SAU; 6 Pathology and Laboratory Medicine, King Saud Bin Abdulaziz University for Health Sciences College of Medicine, Jeddah, SAU

**Keywords:** down-klinefelter syndrome, klinefelter, down, syndrome, chromosome, aneuploidy

## Abstract

Aneuploidy is a category of chromosomal abnormalities involving a numerical abnormality of the chromosomes. The most common type seen in live-born babies is trisomy. Double aneuploidy that leads to trisomy of two different chromosomes occurs due to accidental meiotic nondisjunction events; both can have the same or a different parental origin. Other frequently found double aneuploidies include 48,XXX,+21; 48,XXY,+18, and 48,XXX,+18. Here, we report the case of double aneuploidy (Down-Klinefelter syndrome) in a Saudi newborn with the clinical features of Down syndrome, along with hypothyroidism and congenital heart disease, who was admitted to our neonatal intensive care unit. To our knowledge, this is the first case of its kind reported from the Kingdom of Saudi Arabia.

## Introduction

The incidence of Down syndrome is approximately 1 in 732 newborns in the United States [1]. The risk of having a child with Down syndrome increases with maternal age [2]. In Saudi Arabia, the prevalence of Down syndrome is 6.6 per 10,000 children [3].

Trisomy 21 is present in around 94% of people with Down syndrome because of meiotic nondisjunction, the failure of homologous chromosomes or sister chromatids to separate during cell division. This nondisjunction is characterized by various dysmorphic features, such as a round face, short neck, low-set ears, upslanting eyes, depressed nasal bridge, and micrognathia with a protruding tongue.

Klinefelter syndrome is a chromosomal disorder characterized by an extra X chromosome in male cells [4]. Normal cells in males contain 46 chromosomes (46,XY karyotype), and the extra X chromosome results in a total of 47 chromosomes (47,XXY karyotype). Klinefelter syndrome is the most common human sex chromosome disorder worldwide, with an estimated prevalence of approximately 1 in 500 males [5].

Aneuploidy is the category of chromosome mutations related to numerical abnormalities of the chromosomes. It can involve somatic chromosomes (e.g., 13, 18, or 21) and sex chromosomes, and each mutation can manifest as monosomy, trisomy, or even tetra or pentasomy [6]. Double aneuploidy (Down-Klinefelter syndrome) is rare, with an estimated incidence of 0.4-0.9 cases per 10,000 male births and 11.7 cases per 10,000 cases of trisomy 21 [7]. It is characterized by the presence of a third copy of chromosome 21 plus an extra X chromosome, resulting in 48 chromosomes (48,XXY,+21). The rate of double aneuploidy (Down-Klinefelter syndrome) in the same individual is approximately 0.098% [8].

The first case of double aneuploidy (Down-Klinefelter syndrome) was reported in 1959 by Ford et al. [9], and many cases have been reported since then. In this report, we present a rare case of double aneuploidy in a Saudi boy. To our knowledge, our case is the first to be reported from Saudi Arabia.

## Case presentation

An early-term baby boy was born to a 42-year-old Saudi female (gravida 16, para 14 with one abortion) at 37+1 weeks gestational age. The mother was booked at our hospital. She was known to have hypothyroidism (on medication) and gestational diabetes mellites (on insulin). Antenatal ultrasound of the infant showed polyhydramnios and small kidneys bilaterally, with no other anomalies. All maternal antenatal serology test results were unremarkable. Her family history was positive for a first-degree cousin. One sibling had a cleft lip and palate. The family had no known history of chromosomal abnormalities or other syndromes.

The mother had a history of prolonged rupture of the membrane for 18 hours before delivery. She was taken for an emergency cesarean section due to a breech presentation. At the time of delivery, Apgar scores were 7 and 9 at one and five minutes, respectively. Immediately after birth, the infant developed respiratory distress and desaturation and was transferred to the neonatal intensive care unit (NICU) and placed on a low-flow nasal cannula with a fraction of inspired oxygen of 25%.

**Figure 1:**
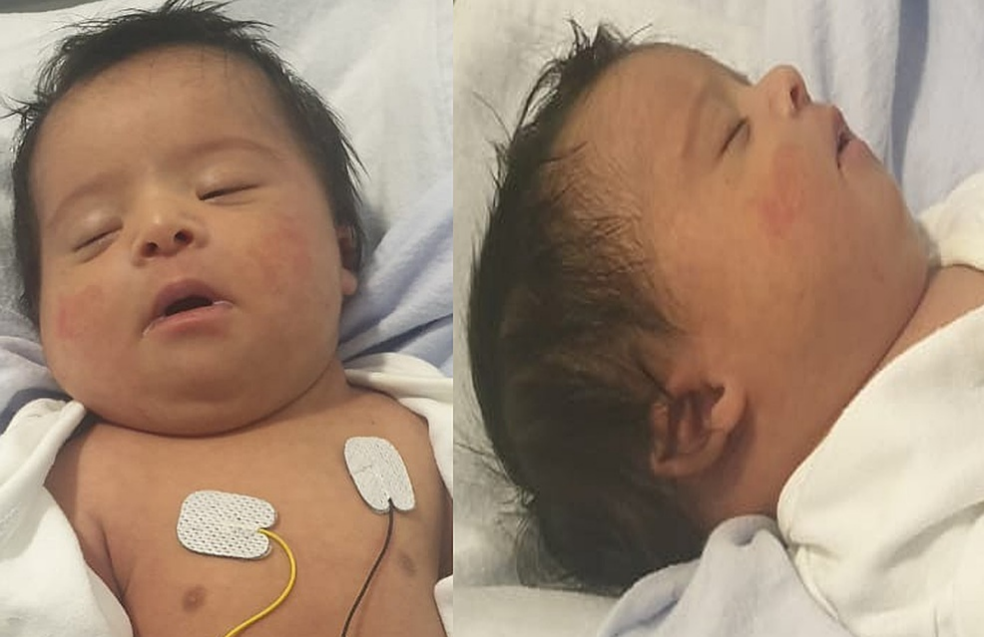
Facial features of the newborn: a broad forehead, upslanting eyes, infraorbital crease, hypertelorism, depressed nasal bridge, flat philtrum, and low-set and malformed ears with attached ear pinnae.

The infant’s birth weight was 3,490 g (20th percentile), length 53 cm (55th percentile), and head circumference 34 cm (6th percentile). His vital signs were stable (temperature, 36.8°C; heart rate, 152 beats/minute; respiratory rate, 62 breaths/minute; blood pressure, 55/30 mmHg; and oxygen saturation, 94% on a 25% fraction of inspired oxygen). An initial newborn physical examination revealed dysmorphic features in the form of a broad forehead, upward slanting eyes, infraorbital crease, hypertelorism, depressed nasal bridge, flat philtrum, low-set malformed ears with attached ear pinnae, and micrognathia with a large tongue and high arched palate. The neck was short and webbed. No simian crease or clinodactyly was noted (Figure 1).

The infant had mild hypotonia, with intact neonatal reflexes. A soft systolic ejection murmur of grade 2/6 was heard, with good perfusion and capillary refill. The abdomen was soft and lax, with no organomegaly. Both testes were descended, and the anus was normal. A partial sepsis evaluation was initiated, and the infant was administered ampicillin and gentamicin.

A chest X-ray showed a small lung volume, cardiomegaly, and increased bronchovesicular markings. An echocardiogram showed moderate patent ductus arteriosus (PDA) and two ventricular septal defects (anterior and posterior). Renal ultrasound showed bilateral small kidneys with no hydronephrosis. The thyroid function test showed a mild increase in thyroid-stimulating hormone TSH (13.8 mIU/L) with normal free T4 (20.7 pmol/L).

**Figure 2:**
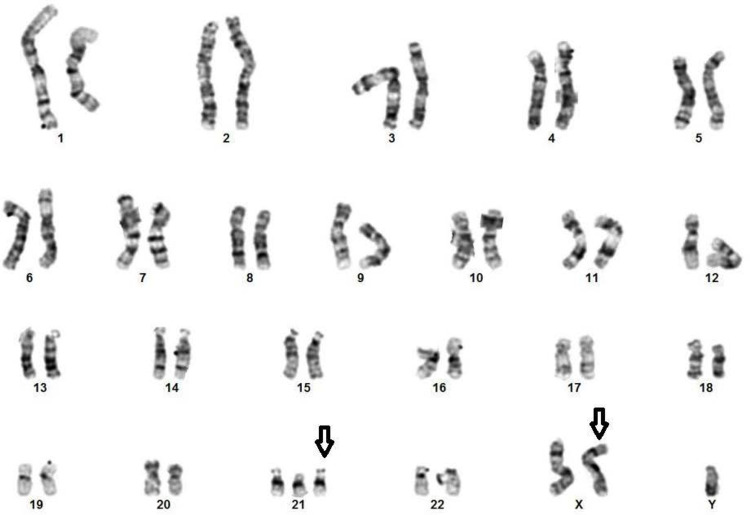
G-banded karyotype shows a double aneuploidy (48,XXY,+21), Down-Klinefelter syndrome. The arrows indicate the extra chromosome and X chromosome.

The chromosomal analysis confirmed 48,XXY,+21, with an abnormal male chromosome complement in all cells examined, along with the presence of an extra X chromosome and an extra chromosome 21 (trisomy 21) (Figure 2).

This result was consistent with the diagnosis of a rare double aneuploidy, Down-Klinefelter syndrome.

The baby stayed in the NICU in stable condition, and the respiratory support was discontinued by the age of three days and full feeding was initiated. He was discharged at the age of nine days in stable condition, with outpatient clinic follow-up with multiple subspecialties (genetics, pediatric endocrine, pediatric ENT, and pediatric cardiologist). He subsequently had three admissions to the pediatric intensive care unit due to bronchiolitis. The first was due to rhinovirus/enterovirus and parainfluenza virus type 4 pneumonia, for which he required mechanical ventilation for a few days. He was discharged in stable condition after 21 days. He was admitted again due to respiratory infection secondary to pertussis and required continuous positive airway pressure for a few days, and was then weaned to nasal cannula oxygen. He was discharged on home oxygen after approximately one month. He was admitted again due to pneumonia with congestive heart failure and required intubation and mechanical ventilation. During this admission, PDA ligation was done, which improved his heart failure with concomitant medical therapy, following which he was weaned off oxygen. Postoperatively, he developed stridor due to left vocal cord palsy and subglottic stenosis. The patient improved clinically and was discharged home after 38 days with scheduling for a multidisciplinary team clinical follow-up. He is currently doing well, as per his last outpatient visit, with no active concerns.

## Discussion

Trisomy 21, or Down syndrome, is one of the best-recognized and most common chromosome disorders caused by the presence of all or part of a third copy of chromosome 21 [10]. It is the single most common genetic cause of mental retardation [11]. This extra copy of the chromosome changes how the neonate’s body and brain develop, resulting in multisystem conditions that include but are not limited to neurological diseases (e.g., Alzheimer’s disease), cardiovascular system defects (e.g., atrioventricular septal defect), gastrointestinal system defects (e.g., duodenal atresia), endocrine diseases (e.g., hypothyroidism), and hematologic diseases (e.g., leukemia) [12].

The most prevalent sex chromosomal condition linked to male hypogonadism and infertility is Klinefelter syndrome. It is caused by the presence of an additional X chromosome in a male. 47,XXY is the most common karyotype, accounting for 80-90% of all cases. Mosaicism (46,XY/47,XXY; 46,XY/48,XXXY; and 47,XXY/48,XXXY) is observed in about 10% of cases [13]. It is characterized by small testes, a small penis, a taller than average height, and behavioral concerns. In late puberty, affected individuals develop gynecomastia, decreased facial and body hair, and azoospermia [4].

Double aneuploidy with Down-Klinefelter syndrome, as seen in our case, usually presents in the neonatal period with the clinical features of Down syndrome alone, while features characteristic of Klinefelter syndrome appear later.

The prenatal mortality rate of Down-Klinefelter syndrome has not been extensively studied. One study reported two miscarriages out of 10 cases of prenatally diagnosed Down-Klinefelter syndrome, with a mortality rate of 20%. The study reported that the risk for 48,XXY,+21 was age-dependent, with a mean maternal age of 33 years and a mean paternal age of 38 years [8]. However, the parents of our case were older, as the mother was 42 and the father was 50 years old.

Only some of the reported cases of 48,XXY,+21 are associated with cardiac defects [7,8,14,15]. Most cases present with typical features of Down syndrome but without recurrent chest infection or congenital heart, thyroid, or digestive problems. They also have normal stature [16]. Another study described a patient with no cardiovascular or digestive system anomalies but with a history of recurrent chest infection and short stature [17]. A case was also reported of an infant who had undescended testis and clinical features of Down syndrome associated with hypothyroidism [18]. Another case reported a child with double aneuploidy associated with a double aortic arch [15].

Our case report of Down-Klinefelter syndrome was characterized by the clinical features of Down syndrome and accompanying heart defects, including PDA and ventricular septal defects, as well as hypothyroidism and recurrent chest infection. To our knowledge, including our case, only 67 cases of double trisomy with a 48,XXY,+21 chromosome pattern have been reported (Table 1), but only 12 of the 67 cases had congenital heart disease (Table 2).

**Table 1:** Previously reported cases of Down and Klinefelter syndrome.

Karyotype	Phenotype	Reference
48,XXY,+21	Klinefelter/Down syndrome	[1]
48,XXY,+21	Klinefelter/Down syndrome	[19]
48,XXY,+21	Klinefelter/Down syndrome	[20]
48,XXY,+21	Klinefelter/Down syndrome	[21]
48,XXY,+21	Klinefelter/Down syndrome	[22]
48,XXY,+21	Klinefelter/Down syndrome	[23]
48,XXY,+21	Klinefelter/Down syndrome	[24]
48,XXY,+21	Klinefelter/Down syndrome	[25]
48,XXY,+21	Klinefelter/Down syndrome	[26]
48,XXY,+21	Klinefelter/Down syndrome	[27]
47,XXY/48,XXY,+21	Klinefelter/Down syndrome-mosaic	[28]
48,XXY,+21	Klinefelter/Down syndrome	[29]
48,XXY,+21	Klinefelter/Down syndrome	[14]
48,XXY,+21	Klinefelter/Down syndrome	[30]
48,XXY,+21	Klinefelter/Down syndrome	[31]
48,XXY,+21	Klinefelter/Down syndrome	[32]
48,XXY,+21	Klinefelter/Down syndrome	[33]
48,XXY,+21	Klinefelter/Down syndrome	[34]
48,XXY,+21	Klinefelter/Down syndrome	[8]
48,XXY,+21	Klinefelter/Down syndrome	[15]
48,XXY,+21	Klinefelter/Down syndrome	[7]
48,XXY,+21	Klinefelter/Down syndrome	[14]
48,XXY,+21	Klinefelter/Down syndrome	[35]
48,XXY,+21	Klinefelter/Down syndrome	[36]

**Table 2:** Reported cases of Down and Klinefelter syndrome with congenital heart disease.

Number	Congenital heart disease	Reference
1	Moderate patent ductus arteriosus, two ventricular septal defects (anterior and posterior), and CoA	Our case
2	Atrial septal defect, ventricular septal defect, and patent ductus arteriosus	[37]
3	Complex congenital heart disease with an ostium secundum atrial septal defect, enlarged right ventricle, and mild tricuspid valve regurgitation	[14]
4	Large atrial septal defect and ventricular septal defect with patent ductus arteriosus, pulmonary hypertension, and mild tricuspid regurgitation	[7]
5	Twins: first, cardiac anomalies; second, an open ductus and a septum anomaly	[21]
6	Cardiac anomalies	[38]
7	Mild aortic stenosis with possible coexistent pulmonary stenosis	[39]
8	A surcharge of the right auricle and ventricle compatible	[40]
9	A systolic murmur, and generalized cyanosis developed during exercise	[41]
10	Atrioventricular septal defect with pulmonary stenosis	[42]
11	A small atrial septal defect (secundum type) and a double aortic arch	[15]
12	Atrioventricular canal defect	[8]

## Conclusions

The described case of double trisomy with 48,XXY,+21 chromosomes could be a challenging diagnosis as the only manifestation at birth are features typical of Down syndrome. In our case, the karyotype incidentally showed an additional X chromosome. This finding is important for counseling parents about the possible additional presentations of Klinefelter syndrome later during puberty.

In the presence of typical features of Down syndrome, we highly recommend proceeding with chromosomal analysis in each case of phenotypic Down syndrome. Cytogenetic testing would confirm the diagnosis. Moreover, excluding additional, albeit rare, chromosomal abnormalities, such as double aneuploidies, is important.

## References

[1] Sherman SL, Allen EG, Bean LH, Freeman SB (2007). Epidemiology of Down syndrome. Ment Retard Dev Disabil Res Rev.

[2] Morris JK, Mutton DE, Alberman E (2002). Revised estimates of the maternal age specific live birth prevalence of Down's syndrome. J Med Screen.

[3] AlSalloum A, El Mouzan MI, AlHerbish A, AlOmer A, Qurashi M (2015). Prevalence of selected congenital anomalies in Saudi children: a community-based study. Ann Saudi Med.

[4] Groth KA, Skakkebæk A, Høst C, Gravholt CH, Bojesen A (2013). Clinical review: Klinefelter syndrome--a clinical update. J Clin Endocrinol Metab.

[5] Los E, Ford GA (2021). Klinefelter syndrome. https://www.ncbi.nlm.nih.gov/books/NBK482314/.

[6] Shen Z, Zou CC, Shang SQ, Jiang KW (2012). Down-Klinefelter syndrome (48,XXY,+21) in a child with congenital heart disease: case report and literature review. Intern Med.

[7] Jeanty C, Turner C (2009). Prenatal diagnosis of double aneuploidy, 48,XXY,+21, and review of the literature. J Ultrasound Med.

[8] Kovaleva NV, Mutton DE (2005). Epidemiology of double aneuploidies involving chromosome 21 and the sex chromosomes. Am J Med Genet A.

[9] Ford C, Jones KW, Miller OJ, Mittwoch U, Penrose LS, Ridler M, Shapiro A (1959). The chromosomes in a patient showing both mongolism and the Klinefelter syndrome. Lancet.

[10] Holmes G (2014). Gastrointestinal disorders in Down syndrome. Gastroenterol Hepatol Bed Bench.

[11] Rachidi M, Lopes C (2007). Mental retardation in Down syndrome: from gene dosage imbalance to molecular and cellular mechanisms. Neurosci Res.

[12] Akhtar F, Bokhari SR (2022). Down syndrome (trisomy 21). https://pubmed.ncbi.nlm.nih.gov/30252272/.

[13] Catović A (2008). Phenotype manifestations of polysomy X at males. Bosn J Basic Med Sci.

[14] Shu X, Zou C, Shen Z (2013). Double aneuploidy 48,XXY,+21 associated with a congenital heart defect in a neonate. Balkan J Med Genet.

[15] Gerretsen MF, Peelen W, Rammeloo LA, Koolbergen DR, Hruda J (2009). Double aortic arch with double aneuploidy--rare anomaly in combined Down and Klinefelter syndrome. Eur J Pediatr.

[16] Hou JW, Wang TR (1996). Double aneuploidy with Down's-Klinefelter's syndrome. J Formos Med Assoc.

[17] Zaki MS, Kamel AA, El-Ruby M (2005). Double aneuploidy in three Egyptian patients: Down-Turner and Down-Klinefelter syndromes. Genet Couns.

[18] Babu Rao V, Ghosh K (2003). Combined Down and Klinefelter syndrome. Indian Pediatr.

[19] Harnden DG, Miller OJ, Penrose LS (1960). The Klinefeltermongolism type of double aneuploidy. Ann Hum Genet.

[20] Lanman JT, Sklarin BS, Cooper HL, Hirschhorn K (1960). Klinefelter's syndrome in a ten-month-old mongolian idiot: report of a case with chromosome analysis. N Engl J Med.

[21] Hustinx TW, Eberle P, Geerts SJ, Brink T, Woltring LM (1961). Mongoloid twins with 48 chromosomes (AA plus A21XXY). Ann Hum Genet.

[22] Gelderen V, Hustinx T (1961). Combination of Klinifelter’s syndrome and mongol‐ism. Ned Tij Genet.

[23] Al-Awadi SA, Naguib KK, Bastaki L (1998). Down syndrome in Kuwait: recurrent familial trisomy 21 in siblings. Downs Syndr Res Pract.

[24] Milcou S, Maicanesco M (1963). [Klinefelter's syndrome associated with mongolism and bilateral testicular ectopy]. Pathol Biol.

[25] Courtbrown WM, Harnden DG, Jacobs PA, Maclean N, Mantle DJ (1964). Abnormalities of the sex chromosome complement in man. Memo Med Res Counc.

[26] Pfeiffer RA (1964). [Double aneuploidy (trisomy 21 and XXY) in an infant]. Arch Kinderheilkd.

[27] Hamerton JL, Briggs SM, Giannelli F, Carter CO (1961). Chromosome studies in detection of parents with high risk of second child with Down's syndrome. Lancet.

[28] Yamaguchi T, Hamasuna R, Hasui Y, Kitada S, Osada Y (1989). 47,XXY/48,XXY,+21 chromosomal mosaicism presenting as hypospadias with scrotal transposition. J Urol.

[29] Iliopoulos D, Poultsides G, Peristeri V, Kouri G, Andreou A, Voyiatzis N (2004). Double trisomy (48,XXY,+21) in monozygotic twins: case report and review of the literature. Ann Genet.

[30] Cyril C, Neha C, Jegatheesan T (2005). Case report-Down syndrome child with 48, XXY,+ 21 karyotype. Ind J Hum Genet.

[31] Glass IA, Li L, Cotter PD (2006). Double aneuploidy (48,XXY,+21): molecular analysis demonstrates a maternal origin. Eur J Med Genet.

[32] Akbas E, Soylemez F, Savaoglu K, Hallioglu O, Balci S (2008). A male case with double aneuploidy (48, XXY,+ 21). Genet Couns.

[33] Karaman A, Kabalar E (2008). Double aneuploidy in a Turkish child: Down-Klinefelter syndrome. Congenit Anom (Kyoto).

[34] Biselli JM, Machado FB, Zampieri BL (2009). Double aneuploidy (48,XXY,+21) of maternal origin in a child born to a 13-year-old mother: evaluation of the maternal folate metabolism. Genet Couns.

[35] Mishra SR, Bisht JS, Kumar M (2014). Double aneuploidy 48, XXY,+ 21 in a fetus with congenital abnormalities. Der Pharm Lett.

[36] Pinti E, Lengyel A, Fekete G, Haltrich I (2020). What should we consider in the case of combined Down- and 47,XY,+i(X)(q10) Klinefelter syndromes? The unique case of a male newborn and review of the literature. BMC Pediatr.

[37] Bozdogan ST, Bisgin A (2017). A rare double aneuploidy case (Down-Klinefelter). J Pediatr Genet.

[38] De Grouchy J, Emerit I, De Gennes JL, Vernant P (1965). [Klinefelter's syndrome in a 6-year-old trisomy-21 boy]. Presse Med.

[39] Hecht F, Nievaard JE, Duncanson N (1969). Double aneuploidy: the frequency of XXY in males with Down's syndrome. Am J Hum Genet.

[40] Erdtmann B, de Freitas AA, de Souza RP, Salzano FM (1971). Klinefelter's syndrome and G trisomy. J Med Genet.

[41] Efinski D, Duma H, Apostolovski B, Sofijanov N, Ristevski B, Darkovski S (1974). Klinefelter's and Down's syndrome in an adolescent with abnormal EEG. Clin Genet.

[42] Akbas E, Soylemez F, Savasoglu K, Halliogluand O, Balci S (2008). A male case with double aneuploidy (48,XXY,+21). Genet Couns.

